# Investigating the predictive rate of protection motivation theory construct considering the preventive behaviors of drowning among parents of elementary school students 

**Published:** 2022-01

**Authors:** Leila Keikavoosi-Arani, Elham Ehsani-Chimeh

**Affiliations:** ^*a*^ Assistant Professor of Healthcare Services Management, Department of Healthcare Services Management, School of Health, Alborz University of Medical Sciences, Karaj, Iran.; ^*b*^ Assistant Professor of Healthcare Services Management, National Institute for Health Research, Tehran University of Medical Sciences, Tehran, Iran.

## Abstract

**Background:**

Despite the high prevalence of drowning in the world, global estimates may underestimate the real public health problem of drowning. The aim of this study was to determine the predictive extent of protection motivation model constructs in drowning prevention behaviors from the perspective of parents of elementary school students.

**Methods:**

In this descriptive-analytical study, 340 parents of primary school students in the west of Tehran province were studied. Sampling was done by cluster sampling method. First, among the government schools in the west of Tehran province, 4 schools were randomly selected (2 girl’s school and 2 boys' schools with a population of approximately 300 students in the elementary school) (N = 1200). Based on Krejcie and Morgan table, the sample size was estimated 291 people. Considering the 20% drop, the sample number was 349. The data collection tool was a researcher-made questionnaire, the reliability and validity of which were assessed. Data were analyzed using SPSS19 software and Pearson correlation test and linear regression.

**Results:**

The mean and standard deviation of the age of the parents was 32.55± 7.50. The present research findings show that this model explains 67% of the variance of drowning marine trauma prevention behaviors. In this model, perceived severity (ß =0.157 and P = 0.011), fear (ß =0.150 and P = 0.011), perceived cost (ß- =0.153 and P = 0.019) and behavior (ß =0.213 and P = 0.004) significantly predicted drowning prevention behavior.

**Conclusions:**

Due to the greater correlation between protection motivation model constructs with the observance of drowning marine trauma prevention recommendations, the design and implementation of educational programs by school health educators are effective for increasing the motivation of primary school students.

**Keywords:**

Drowning, Protection Motivation Model, Elementary School, Iran

